# Supplementary figures and images for: SIRPα - CD47 axis regulates dendritic cell-T cell interactions and TCR activation during T cell priming in spleen
Source: PLoS One. 2022 Apr 12;17(4):e0266566. doi: 10.1371/journal.pone.0266566 (PMC9004769; doi:10.1371/journal.pone.0266566)

Supporting Figure 1

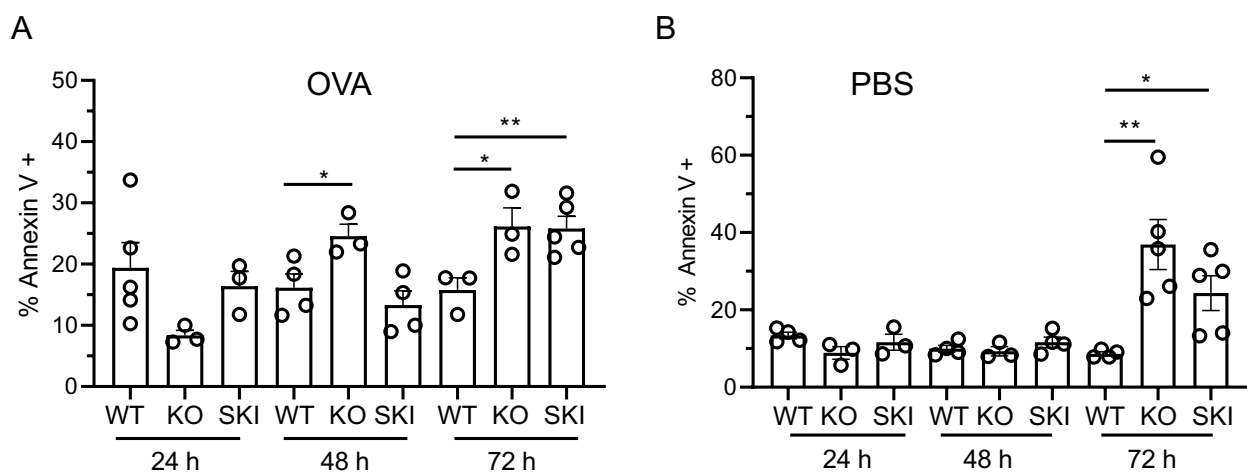

Supplement: S1 Fig — (A,B) WT, Cd47KO (KO) and SKI naive OTII T cells were labeled with violet dye and transferred i.p. into WT, KO and SKI recipient mice, respectively. After 24 hr mice were injected i.p. with OVA (A) or PBS (B) and spleens harvested after 24, 48 or 72 h. CD4+ OTII T cells were assessed for Annexin V and PI staining by flow cytometry. (PDF) [file pone.0266566.s001.pdf]

## Supporting Figure 2

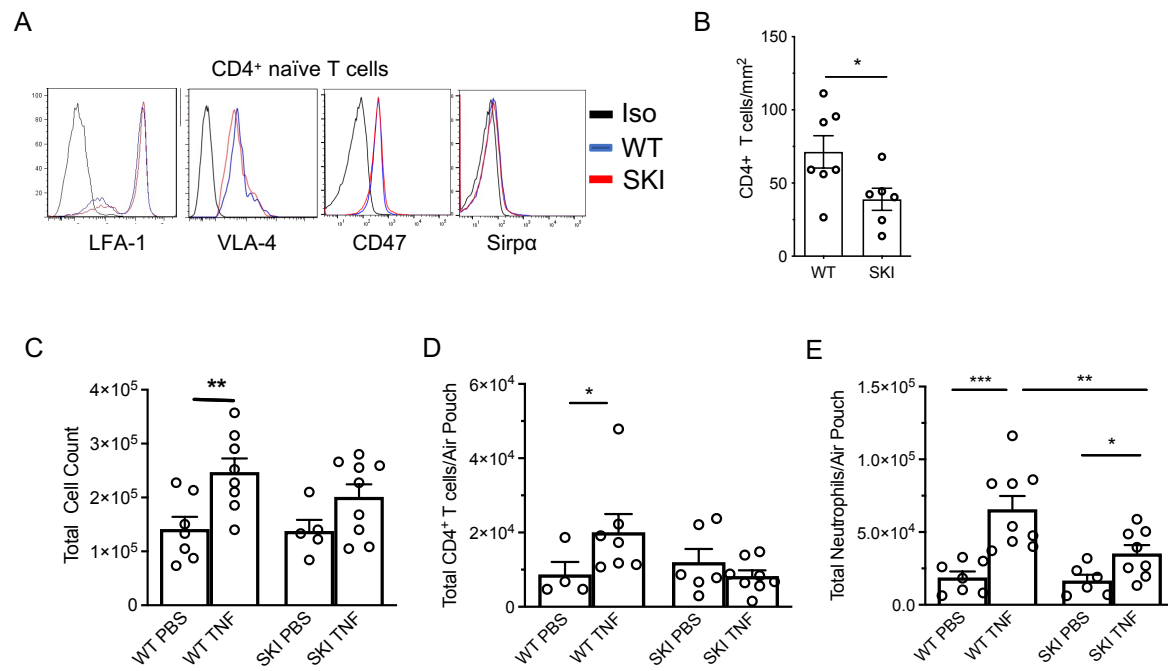

Supplement: S2 Fig — Data are representative of 3 WT and SKI mice. (B) T cell arrest on ICAM-1 coimmobilized with 200 ng/100 μL of SDF-1α (CXCL12) at an estimated laminar shear stress of 1.0 dynes/cm2. n = 2 separate experiments, replicates performed in triplicate. * p<0.05 by Student t test. (C-E) WT and SKI mice had increased immune cell infiltrates in response to rmTNF-α in a dermal air pouch model of recruitment. SKI mice had significantly reduced influx of CD4+ T cells and neutrophils compared to WT mice. N = 3–5 mice per group in 2 separate experiments *p<0.05, **p<0.01, *** P< 0.005. (PDF) [file pone.0266566.s002.pdf]

### Supporting Figure 3

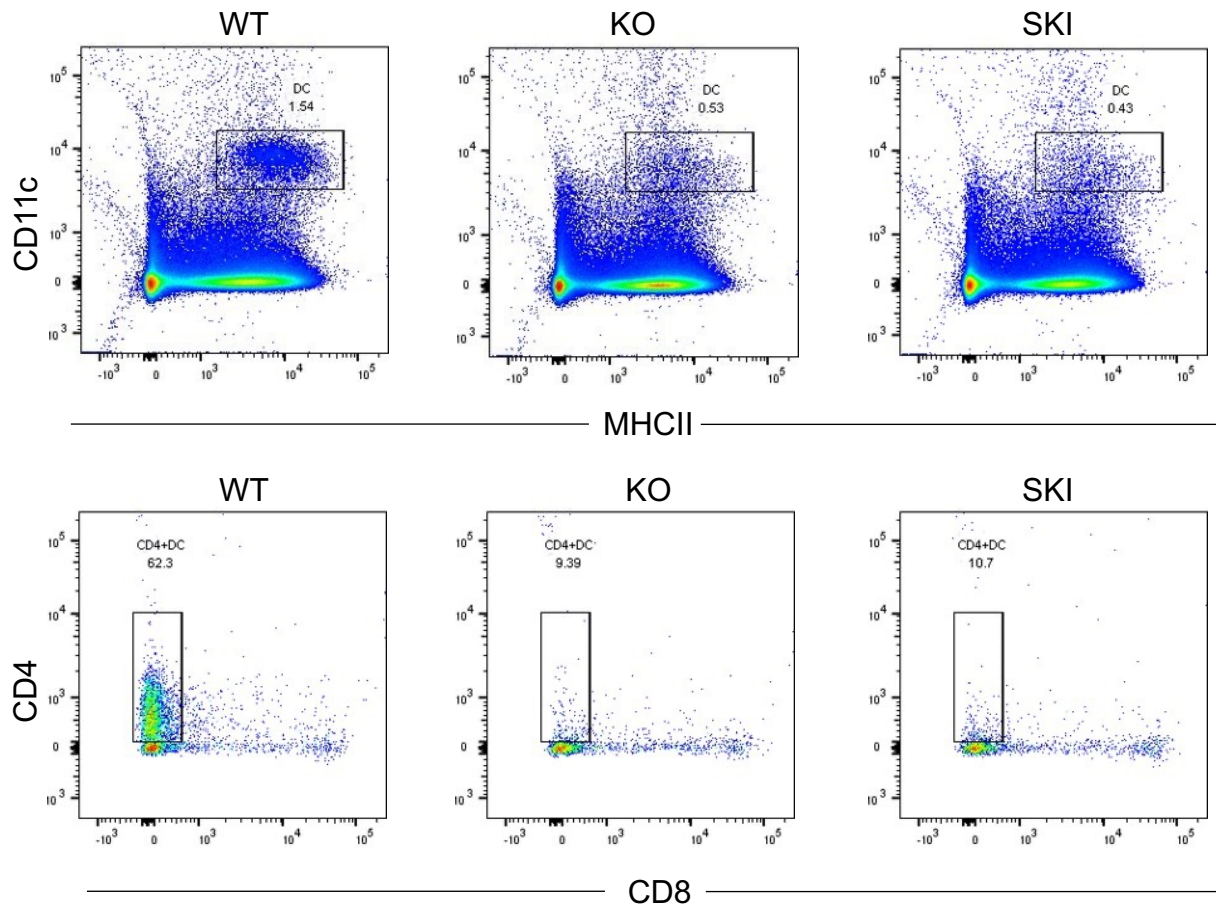

Supplement: S3 Fig — Representative plots of CD45+, CD11c+ MHCIIhi DC and CD4+ DC in WT (62.3%), Cd47KO (9.39%) and SKI (10.7%) mice as determined by flow cytometry. (PDF) [file pone.0266566.s003.pdf]

## Supporting Figure 4

WT

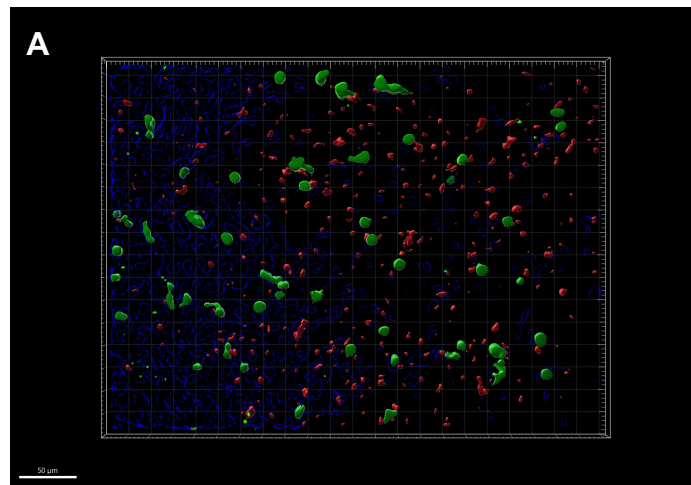

*Cd47*KO

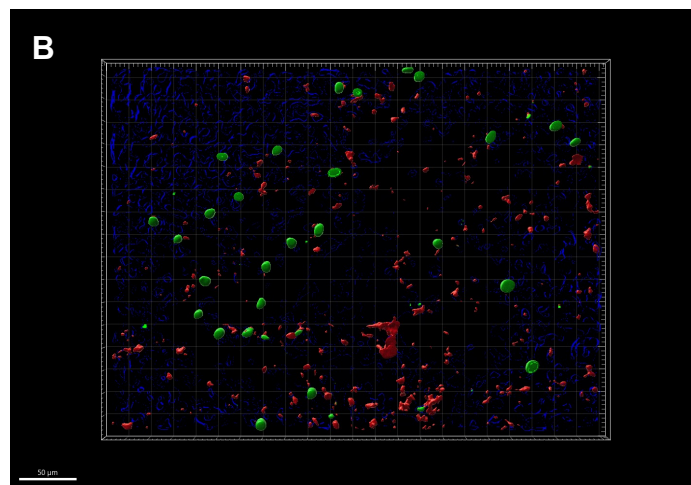

SKI

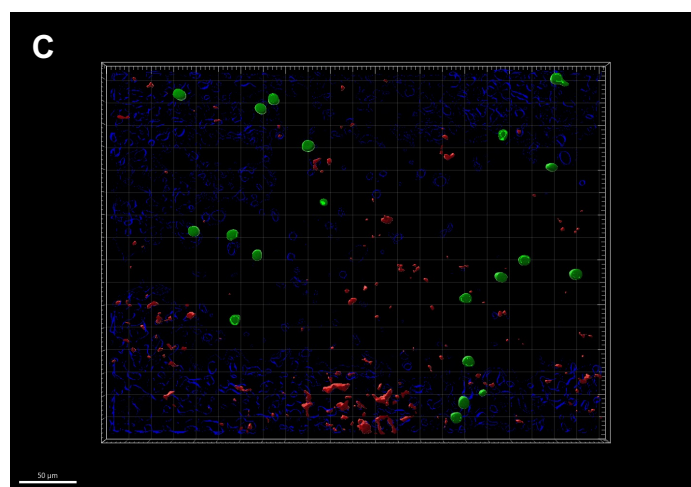

Supplement: S4 Fig — A–C. Representative three-color images of splenic sections were imported into IMARIS software, surface renderings were performed to identify B cell zones (blue cells) surrounding T cells zones that contain transferred CD4+ OTII T cells (green) and endogenous CD11c stained DCs (red). Computation of distances of DCs from CFSE cells were determined by IMARIS from 11 Cd47KO, 11 SKI and 8 WT mouse samples. Calibration bars = 50μm. Data are representative images of different T cell zones in spleen analyzed for 3 mice per group. (PDF) [file pone.0266566.s004.pdf]
