# Supplementary material for: SIRPα - CD47 axis regulates dendritic cell-T cell interactions and TCR activation during T cell priming in spleen
Source: PLoS One. 2022 Apr 12;17(4):e0266566. doi: 10.1371/journal.pone.0266566 (PMC9004769; doi:10.1371/journal.pone.0266566)
Supplement: Attachment — Submitted filename: Reply to Reviewers.docx [file pone.0266566.s005.docx]

Reviewers' comments:
Reviewer's Responses to Questions

**Comments to the Author**

1. Is the manuscript technically sound, and do the data support the conclusions?

The manuscript must describe a technically sound piece of scientific research with data that supports the conclusions. Experiments must have been conducted rigorously, with appropriate controls, replication, and sample sizes. The conclusions must be drawn appropriately based on the data presented.

Reviewer #1: Yes

Reviewer #2: Partly

2. Has the statistical analysis been performed appropriately and rigorously?

Reviewer #1: Yes

Reviewer #2: Yes

3. Have the authors made all data underlying the findings in their manuscript fully available?

The [PLOS Data policy](http://www.plosone.org/static/policies.action#sharing) requires authors to make all data underlying the findings described in their manuscript fully available without restriction, with rare exception (please refer to the Data Availability Statement in the manuscript PDF file). The data should be provided as part of the manuscript or its supporting information or deposited to a public repository. For example, in addition to summary statistics, the data points behind means, medians and variance measures should be available. If there are restrictions on publicly sharing data—e.g., participant privacy or use of data from a third party—those must be specified.

Reviewer #1: Yes

Reviewer #2: Yes

4. Is the manuscript presented in an intelligible fashion and written in standard English?

PLOS ONE does not copyedit accepted manuscripts, so the language in submitted articles must be clear, correct, and unambiguous. Any typographical or grammatical errors should be corrected at revision, so please note any specific errors here.

Reviewer #1: Yes

Reviewer #2: Yes

 5. Review Comments to the Author

Please use the space provided to explain your answers to the questions above. You may also include additional comments for the author, including concerns about dual publication, research ethics, or publication ethics. (Please upload your review as an attachment if it exceeds 20,000 characters).

REPLY: We thank the Editors and both Reviewers for their helpful comments and suggestions to clarify and improve the presentation of our results and the conclusions in the manuscript. All responses are in red text. The revised manuscript used Word “tracking” to identify all revisions.

**Reviewer #1:**

Autio A and Wang H et al., report on the role of the SIRPa-CD47 axis in regulating DC-T cell interactions. Well-written manuscript extending the team’s previous work on CD47. Using several genetic mice tools, authors show that CD47 is necessary for CD4 T cell activation and proliferation in vivo. Because DC-T cell interactions in vitro are no affected and the homing of T cells to the secondary lymphoid tissues is also similar, authors test the hypothesis that SIRPa-CD47 is required for the migration of activated DCs carrying the antigens do the secondary lymphoid organs. This is shown using multiplexed BMDC migration assay in vivo and quantifying DC numbers in the spleen.

Major comments:

- Greater T-DC distance in CD47KO and SKI mice may be because of decreased number of DCs in the spleen due to migration defects as demonstrated in figure 5.
- Experimental details on data in figure 6 need more explanation. Figure 6A legend reads “Four-color immunofluorescence microscopy of transferred CFSE+ OTII T cells, CD11c+, B cell follicles and CD169+ marginal zone macrophages within the splenic white pulp”. How were the cells were injected i.v., i.p, or hock?

REPLY: We thank the Reviewer for helpful comments to clarify and improve our manuscript.

- We suggest the combination of defects in FRC reported previously (1, 2) and reduction in numbers and impaired migration of splenic CD4^+^ cDC cells caused the greater distances between T cells and DCs in SKI and CD47KO mice. We have revised the Results (pg 21, lines 469-474) and Discussion (pg 23 lines 514-517 and 565-581) to emphasize our revised conclusions.
- The experiments shown in Figure 6 were generated using i.v. injection of T cells. Briefly, CFSE-labeled WT OTII T cells (4x10^6^) were i.v. injected into KO, SKI and WT recipient mice. Twenty-four hrs later, spleens were harvested and processed into frozen blocks (3 mice/group, n=2 separate experiments.

Minor comment: Image quality is lost in transformation.

REPLY: This comment is not specific, so we assume that the Reviewer referred to immunofluorescence images in Figure 6A, 6B and S. Fig 4. The images presented were obtained with the greatest resolution possible with our image capture equipment. The conversion to PDF provided to Reviewers are lower quality than the images.tiff submitted to the journal.

**____________________**

**Reviewer #2:**

In the manuscript by Luscinskas et al., the authors focused on the role of the CD47-SIRPα axis in the antigen-specific DC-T interaction and demonstrated impaired OT-II T cell proliferation in the spleen of CD47 KO or SIRPα KI mice after OVA immunization. The authors also focused on the interaction of T cells and DCs in the spleen and provided the results suggesting the reduced TCR activation in OT-II T cells transplanted into CD47 KO and SIRPα KI mice, as well as the reduced proximity of DCs to T cells in the spleen of these mice. These results suggest the importance of the interaction between CD47 and SIRPα in the context of the antigen-specific immune response, however, some of the data are still immature and inappropriately concluded. Thus, some modification is necessary for the publication to PloS One.

**REPLY:** We thank the Reviewer for the helpful comments and suggestions to improve the content and clarity of the manuscript.

Major comments
1. Figure 5: Since the spleen does not contain lymphatics, it remains unclear whether the reduction of DC migration from skin to the draining lymph node through lymphatics is related to the reduction of DCs in the spleen. In addition, the previous study suggested that the cause of the reduced splenic CD4+ cDCs in CD47 KO or SIRPα KI mice is still unclear and is in part implicated in the activation of the subset, particularly in the upregulation of CCR7, which can promote the migration of DCs (Yi et al. Immunity 2015: PMID 26453377). Thus, there is not enough evidence to support the reduction of splenic CD4+ cDCs is attributable to the impaired migration of BMDCs from CD47KO and SIRPα KI mice, and the claim “dual effect” described in the discussion is not clear from these results. To show the importance of DC migration in these mice, the author should at least demonstrate if the results shown in Figure 5A-C are dependent on the CCR7-CCL19/21 axis or the activation of BMDCs.

**REPLY:** The Reviewer has raised important comments. We address this comment in two separate replies.

**A.** “Comment 1. Figure 5: Since the spleen does not contain lymphatics, it remains unclear whether the reduction of DC migration from skin to the draining lymph node through lymphatics is related to the reduction of DCs in the spleen.”

We believe that the last two sentences in that paragraph (lines 410-413) were not clear and led to the Reviewer’s comment about the cause of DC defective migration. The main point of the paragraph was the reduction in total splenic DCs numbers (Fig 5D) is likely due to the specific reduction in the CD4^+^ cDC subset as demonstrated by Figs 5E and 5F. We apologize for causing confusing.

The underlying mechanism for the reduction in the CD4^+^ DC subset is complex and recently has been investigated by other groups. Saito and colleagues (1, 2) have reported that a reduction in splenic fibroblastic reticular cells (FRC) and their function in splenic T cell marginal zone led to dramatic reduction of IL-7, a key T cell survival factor for T cells (3)), and CCL19 and CCL21, chemokines the bind CCR7 and attract CD4^+^ T cells and CD4^+^ SIRPα^+^ DCs. Thus, the reduction in IL-7, CCL21 and CCL19 was proposed to cause or significantly contribute to reduction of splenic CD4^+^ T cells and CD4^+^ SIRPα^+^ DCs. These Author also reported that the loss of FRC could be explained by a reduction in TNF-α receptor ligands produced by bone marrow derived SIRPα^+^ DCs that failed to enter the spleen in KO and SKI mice (2). These results have been incorporated into the Introduction (pg 4, lines 70-85), Results (pg 21, lines 469-474) and the Discussion (pgs 23, lines 514-518, and pg 25, lines 564-581).

**B.** To show the importance of DC migration in these mice, the author should at least demonstrate if the results shown in Figure 5A-C are dependent on the CCR7-CCL19/21 axis or the activation of BMDCs.

**REPLY:** CCR7 expression and the activation status of BM-DCs has been examined by other groups and the results did not explain the defect in DC migration. First, Hagnerud and colleagues (4) showed that *Cd47*^-/-^ BM-derived DC compared to WT DCs have similar amounts of surface expressed CCR7, CD40, CD86 and CD80 by flow cytometry, however, *Cd47*^-/-^ DC migration was reduced by ~50% to all 3 concentrations (250, 125, 62 ng/ml) of CCL19 in an in vitro transwell assay. Next, Iwamura and coworkers (5) reported that BM-DCs from SKI and WT mice expressed the same amount of CCR7 by flow cytometry and had no defect in migration to a single supraphysiological amount of CCL19 (250 ng). Lower and more physiological concentrations were not tested. The lack of a defect in migration could be explained by the Author’s use of a single supraphysiological amount of CCL19. Thus, the defects in migration seen in BM-DCs from CD47^-/-^ or SKI mice is not due to amount of CCR7 expression or activation status. Finally, in our hands BM-derived DC generated in vitro from WT, *Cd47*KO and SKI mice demonstrate essentially the same enhanced level of activation as measured by surface expression of co-stimulatory molecules CD80 and CD86 after coculture in vitro for 48 hr with OVA and WT CD4^+^ T cells. See figure A on next page. Thus, additional studies are needed to elucidate the molecular and cellular mechanism for loss of splenic CD4^+^ cDCs.

Fig A legend: In vitro BMDCs derived from SKI and *Cd47*KO mice express similar and high amounts of CD80 and CD86. BM-DC from WT, KO and SKI mice were cultured at 37^o^C for 48 h with WT CD4+ OTII T cells in the presence of 0.01, 0.03 or 1 μg of OVA protein or PBS. Cocultures were washed and stained with primary labeled mAb to CD11c, MHC Class II, CD86, CD80. BMDC prepared from WT, SKI and *Cd47*KO expressed similar amounts of activation markers CD80 and CD86 and activated regardless of culture conditions.

Figure A.

*SKI DC*

WT DC

*Cd47^-/-^ DC*

CD80

CD86

CD11c^+^ MHCII^+^

From the literature, several previous studies found the SIRP-CD47 axis played an important role in leukocyte migration, a crucial step for innate and adaptive immunity. For example, SIRPα has been shown to regulate myeloid leukocyte migration in vivo and in vitro (5-9) although the mechanism was not established. On the other hand, our lab has shown that CD47 regulates leukocyte trafficking through its “*in cis*” association with the leukocyte β2 integrin LFA-1 and β1 integrin VLA-4 in T cells, and Mac-1 integrins in neutrophils (10-12). Thus, we propose that loss of CD47 may cause defects in both β2 and/or β1 integrins. Finally, as mentioned in comment 1 above, the marginal T cell zone is defective in FRC cells leading to diminished production of IL-7 for T cells and the chemoattractants CCL21 and CCL19.

2. As most of the data demonstrated in the present study are related to published studies, the data shown in Figure 6G is worthwhile to indicate the presence of the study. Therefore, the authors should precisely show the data with representative FACS plot of APA1^+^ T cells in each group together with isotype-matched control staining.

Reply: We believe the Reviewer is referring to Figure 5G and not Fig 6. As suggested, new panel was added to Figure 5H (pg 20, lines 453-454 and copied below) and it contains representative flow cytometry plots of APA1 mAb staining of splenocytes from OVA immunized WT, SKI and KO recipient mice and PBS treated WT control mice.

SKI:OTII → SKI

Fig 5H

WT:OTII→ WT

KO:OTII → KO

+ OVA *i.p.*

WT:OTII → WT

PBS, *i.p.*

CFSE

APA1

3. In Figure 7, since the number of DC and CD4 T cells is decreased in the T cell zone of CD47 KO or SIRPα KI mice, the distance between them must increase. Thus, this reviewer cannot understand the necessity to measure the distance of these cells in this Figure. To claim the importance of the CD47-SIRPα axis on DC-T proximity, the authors should demonstrate 1) whether the prolonged distance between T cells and DCs is independent of the reduction in the number of these cells in the spleen of CD47 KO or SIRPα KI mice, as well as 2) whether the proximity of these cells is dependent on transferred OVA antigen, particularly in the control mice.

**REPLY:** We appreciate the Reviewer comment and concerns. The two points raised are difficult to address experimentally. We believe assessing proximity between OTII T cells and DCs upon Ag challenge is one additional piece of data that further explains the failure of Ag priming in these mice. Our revised conclusions are that the observed lack of T cell activation and expansion in *Cd47*KO and SKI mice is caused by the reduced number of CD4^+^ DCs in the spleen caused by their reduced migration into lymphoid organs associated with defective and reduced numbers of FRC, and by defects in the survival and migration of CD4^+^ T cells in vivo. We believe that the splenic compartment has additional yet unidentified defects that further impair immune cell motility and migration in the T cell zone of SKI and *Cd47KO* mice. We speculate that in addition to their reduction in number and cytokine production, the FRC do not form proper scaffold structures that create a reticular network of collagen rich matrix in splenic T cell zones. This would reduce efficient distribution of antigens and CCL19 and CCL21 chemokines thus impairing migration and reduce meaningful interactions between DCs and T cells leading to poor Ag priming. In particular in vitro studies have reported that CCL21 associates with reticular fibers to promotes DC haplotaxis and that CCL19 diffuses and promotes chemotaxis (13). Furthermore, CCL19 has been shown to promote dendrite extension in splenic DCs (14). We have revised the text in Results (pg 21, lines 461-469) and Discussion (pg 23 lines 501-513 and 565-575, and 580-582) to emphasize our revised conclusions.

Minor comments
1. “SIRPα-CD47 interactions” in the manuscript title is an overstatement since the authors did not show direct evidence showing the importance of the interaction between CD47 and SIRPα.

REPLY: We agree that the data have not directly focused on the protein-protein interaction of these two molecules, hence we have revised the title to reflect the signaling pathway. **SIRPα - CD47 axis regulates dendritic cell-T cell interactions and TCR activation during T cell priming in spleen**

2. In Figure 2G, the authors should explain or discuss the reason why SKI OT-II T cells did not recover in WT hosts, although T cells are absent for SIRPα.

REPLY: This result was an unexpected finding because flow cytometry staining for SIRPα is below detection in our analysis of splenic CD4^+^ T cells from SKI mice and corroborates multiple published reports. We have added new experimental data as Supplemental Fig. 1A-E and present the data in the Results (page 14, lines 318 – 324) and Discussion (page 24, lines and 530-536). These data are copied below. The data identify a defect in LFA-1 adhesive function. Interestingly, the defects in SKI T cells mirrors our prior data that CD47KO T cells exhibits defects in LFA-1 and VLA-4 adhesive function (10-12).

E

D

C

Sirpα

CD47

Iso

SKI

WT

CD4^+^ naïve T cells

VLA-4

LFA-1

B

A

**Figure legend.** Splenocytes from WT and SKI mice were stained with primary labeled mAb to CD4, LFA-1, VLA-4, CD47 and SIRPα and primary labeled isotype-matched mAb and fluorescence detected by flow cytometry and analyzed by FlowJo software. Data are representative of 3 WT and SKI mice. **(B)** T cell arrest on ICAM-1 coimmobilized with 200 ng/100 μL of SDF-1α (CXCL12) at an estimated laminar shear stress of 1.0 dynes/cm^2^. n= 3 mice per group, each performed in duplicate. * p< 0.05 by Student t Test. (**C-E)** WT and SKI mice had increased immune cell infiltrates in response to TNF-α in a dermal air pouch model of recruitment, however, SKI mice had significantly reduced CD4^+^ T cells and neutrophils compared to WT mice. N= 3 - 5 mice per group in 2 separate experiments *p<0.05, **p<0.01, *** p< 0.005.

3. Regarding the DC-T cell conjugate formation assay shown in Figure 3, the authors should indicate when OVA is supplemented during the culture.

**REPLY:** We have included this data in Fig 3 legend:
For murine conjugate formation, BM derived DCs were labeled in PBS with 0.5 µM Violet dye, and then pre-cultured with Ova (1μg/ml) or PBS for 2 h at 37ºC. CFSE-labeled naïve OTII CD4^+^ T cells (1x10^5^) and violet-labeled BMDCs (1x 10^5^) were mixed in a 1:1 ratio with mAbs, centrifuged for 1 min at 800 rpm, and incubated for 2 h at 37ºC.

4. The legend in Figure 4E is described in 4F and should be replaced with each other.

**REPLY:** Thank you for catching that error, we have corrected Fig. 4 E and F legends.

5. Nishimura et al. (PMID 32438469) recently demonstrated that the differentiation of effector T cells is attenuated in the draining LN of MOG-immunized DC-specific SIRPα deficient mice. The paper could be cited to mention the importance of SIRPα on DCs for the T cell priming.

**REPLY:** We have incorporated that reference into the Introduction (line 79).

**REFERENCES**

1.	Sato-Hashimoto M, Saito Y, Ohnishi H, Iwamura H, Kanazawa Y, Kaneko T, et al. Signal regulatory protein alpha regulates the homeostasis of T lymphocytes in the spleen. J Immunol. 2011;187(1):291-7.

2.	Saito Y, Respatika D, Komori S, Washio K, Nishimura T, Kotani T, et al. SIRPalpha(+) dendritic cells regulate homeostasis of fibroblastic reticular cells via TNF receptor ligands in the adult spleen. Proc Natl Acad Sci U S A. 2017;114(47):E10151-E60.

3.	ElKassar N, Gress RE. An overview of IL-7 biology and its use in immunotherapy. J Immunotoxicol. 2010;7(1):1-7.

4.	Hagnerud S, Manna PP, Cella M, Stenberg A, Frazier WA, Colonna M, et al. Deficit of CD47 results in a defect of marginal zone dendritic cells, blunted immune response to particulate antigen and impairment of skin dendritic cell migration. J Immunol. 2006;176(10):5772-8.

5.	Iwamura H, Saito Y, Sato-Hashimoto M, Ohnishi H, Murata Y, Okazawa H, et al. Essential roles of SIRPalpha in homeostatic regulation of skin dendritic cells. Immunol Lett. 2011;135(1-2):100-7.

6.	Liu Y, Buhring HJ, Zen K, Burst SL, Schnell FJ, Williams IR, et al. Signal regulatory protein (SIRPalpha), a cellular ligand for CD47, regulates neutrophil transmigration. J Biol Chem. 2002;277(12):10028-36.

7.	Alvarez-Zarate J, Matlung HL, Matozaki T, Kuijpers TW, Maridonneau-Parini I, van den Berg TK. Regulation of Phagocyte Migration by Signal Regulatory Protein-Alpha Signaling. PLoS One. 2015;10(6):e0127178.

8.	Barclay AN, Van den Berg TK. The interaction between signal regulatory protein alpha (SIRPalpha) and CD47: structure, function, and therapeutic target. Annu Rev Immunol. 2014;32:25-50.

9.	Fukunaga A, Nagai H, Noguchi T, Okazawa H, Matozaki T, Yu X, et al. Src homology 2 domain-containing protein tyrosine phosphatase substrate 1 regulates the migration of Langerhans cells from the epidermis to draining lymph nodes. J Immunol. 2004;172(7):4091-9.

10.	Azcutia V, Routledge M, Williams MR, Newton G, Frazier WA, Manica A, et al. CD47 plays a critical role in T-cell recruitment by regulation of LFA-1 and VLA-4 integrin adhesive functions. Mol Biol Cell. 2013;24(21):3358-68.

11.	Azcutia V, Bassil R, Herter JM, Engelbertsen D, Newton G, Autio A, et al. Defects in CD4+ T cell LFA-1 integrin-dependent adhesion and proliferation protect Cd47-/- mice from EAE. J Leukoc Biol. 2017;101(2):493-505.

12.	Azcutia V, Kelm M, Luissint AC, Boerner K, Flemming S, Quiros M, et al. Neutrophil expressed CD47 regulates CD11b/CD18-dependent neutrophil transepithelial migration in the intestine in vivo. Mucosal Immunol. 2021;14(2).

13.	Schumann K, Lammermann T, Bruckner M, Legler DF, Polleux J, Spatz JP, et al. Immobilized chemokine fields and soluble chemokine gradients cooperatively shape migration patterns of dendritic cells. Immunity. 2010;32(5):703-13.

14.	Yanagawa Y, Onoe K. CCL19 induces rapid dendritic extension of murine dendritic cells. Blood. 2002;100(6):1948-56.

6. PLOS authors have the option to publish the peer review history of their article ([what does this mean?](https://journals.plos.org/plosone/s/editorial-and-peer-review-process#loc-peer-review-history)). If published, this will include your full peer review and any attached files.


If you choose “no”, your identity will remain anonymous but your review may still be made public.


**Do you want your identity to be public for this peer review?** For information about this choice, including consent withdrawal, please see our [Privacy Policy](https://www.plos.org/privacy-policy).

Reviewer #1: **Yes:** Shivashankar Othy

Reviewer #2: No

[NOTE: If reviewer comments were submitted as an attachment file, they will be attached to this email and accessible via the submission site. Please log into your account, locate the manuscript record, and check for the action link "View Attachments". If this link does not appear, there are no attachment files.]

While revising your submission, please upload your figure files to the Preflight Analysis and Conversion Engine (PACE) digital diagnostic tool, <https://pacev2.apexcovantage.com/>. PACE helps ensure that figures meet PLOS requirements. To use PACE, you must first register as a user. Registration is free. Then, login and navigate to the UPLOAD tab, where you will find detailed instructions on how to use the tool. If you encounter any issues or have any questions when using PACE, please email PLOS at [figures@plos.org](mailto:figures@plos.org). Please note that Supporting Information files do not need this step.

*In compliance with data protection regulations, you may request that we remove your personal registration details at any time.* [*(Remove my information/details)*](https://www.editorialmanager.com/pone/login.asp?a=r)*. Please contact the publication office if you have any questions.*
